# The impact of SLCO1B1 polymorphisms on homocysteine concentrations: evidence for a stronger association in men

**DOI:** 10.3389/fneph.2024.1465380

**Published:** 2025-01-29

**Authors:** Xinyuan Hu, Yanfang Jiang

**Affiliations:** Genetic Diagnosis Center, The First Hospital of Jilin University, Changchun, China

**Keywords:** stroke, homocysteine, SLCO1B1, SNP, GGT

## Abstract

**Background:**

Homocysteine (Hcy) is a risk factor for stroke. In this study, we investigated the relationship between gene polymorphisms, particularly SLCO1B1 and homocysteine (Hcy) concentrations in ischemic stroke patients, with a focus on identifying potential risk factors for elevated Hcy levels.

**Methods:**

A total of 177 ischemic stroke patients, including 99 with single nucleotide polymorphisms (SNPs), underwent pharmacogenomics (PGx) sequencing tests, from September 2022 to November 2023 at the hospital. Logistic regression analysis was used to analyze the relationship between clinical characteristics, SNPs, and Hcy concentrations. In the sub-study, 207 ischemic stroke and 244 non-stroke patients underwent SLCO1B1c.521T>C polymorphism to further demonstrate the role of SLCO1B1c.521T>C polymorphism and homocysteine.

**Results:**

Higher Hcy concentrations were observed in men compared to women. Univariate logistic analysis identified gender, GGT concentrations, B12 concentrations, folic acid concentrations, and SLCO1B1 c.521 CC+CT polymorphism as risk factors for elevated Hcy. Multivariate logistic analysis confirmed that B12 concentrations, folic acid concentrations, and SLCO1B1 CT + CC polymorphism were significant dependent risk factors. In the sub-study, SLCO1B1 CT + CC polymorphism and the male sex were identified as risk factors for Hcy, with the effect of SLCO1B1 polymorphism being more pronounced in men.

**Conclusion:**

Folic acid and vitamin B12 reduce Hcy concentrations, while the SLCO1B1 CT and CC polymorphisms are associated with higher Hcy levels. The impact of SLCO1B1 gene polymorphism on Hcy is notably stronger in the male population, suggesting that genetic factors play a significant role in determining Hcy levels.

## Introduction

Stroke is the second leading cause of disability and death worldwide, and the leading cause of death in China (1, 2). Ischemic stroke, also known as cerebral or cerebral ischemia, affects more people in China than any other type of stroke (3). Important risk factors for stroke include hypertension, diabetes, hyperlipidemia, smoking, and hypertension. High plasma homocysteine (Hcy) is also an important risk factor for stroke. Hcy is a sulfur-containing amino acid that occurs in response to oxidative stress and inflammation and damage to the vascular structure, which promotes the formation of atherosclerosis and rupture of atherosclerotic plaques, increasing the risk of stroke (4). Hcy is formed during the conversion of essential amino acid methionine to cysteine. Factors affecting plasma Hcy level include genetics, nutrition, age, sex, drugs, and disease status (5). A large cross-sectional study of a sample population in the United States demonstrated a nonlinear positive correlation between plasma Hcy levels and stroke prevalence, and no increase in stroke prevalence was observed at a plasma Hcy concentration of 15.3 µmol/L. In addition, there was an L-shaped association between plasma vitamin B6 and folic acid and stroke prevalence, with inflection points for vitamin B6 and folic acid being 65.2 nmol/L and 26 nmol/L, respectively. The relationship between vitamin B12 and stroke prevalence was U-shaped (6). The Chinese Guidelines for Hypertension Management (2018 Revision) list H-type hypertension (Hcy ≥ 10 µmol/L) as a key risk factor for stroke. Many factors affect Hcy concentrations and studies have shown that folic acid deficiency and utilization disorders, vitamin B12 deficiency and genetic factors all affect Hcy concentrations (7). The liver is thought to play an important role in methionine and Hcy metabolism because it has sufficient relevant enzymes to regulate plasma Hcy levels (8). At present, the genes related to Hcy concentrations include MTHFR and MTR (9). In this study, we explored and discussed the relationship between genes and biochemical factors associated with Hcy in stroke patients, in order to provide a new perspective for clinical research.

Drug transporters are mainly distributed in the basal or apical membrane of organs involved in drug biotransformation, such as liver, intestine, brain, and kidney, and play an important role in the pharmacokinetic process (10). Genetic polymorphisms of drug transporters affect drug transport across membranes and contribute to variability in drug disposal and response.

Ischemic stroke patients need to take anti-platelet, lipid-lowering, and antihypertensive drugs after admission. Pharmacogenomics (PGx) has made significant progress in recent years and is now being rapidly applied to clinical treatments. Genetic variants can have a profound effect on an individual’s response to drugs. Knowledge of such variants in patients may help in selecting better treatment regimens, leading to better efficacy, and in some cases, prevention of adverse drug events. Therefore, pharmacogenomic gene detection was performed before medication.

In this study, we included ischemic stroke patients whom underwent pharmacogenomic gene test and Hcy concentration test. We analyzed whether these genes were associated with homocysteine concentrations in patients.

## Methods

From September 2022 to November 2023, we retrospectively enrolled 177 patients (141 men), including those with 99 single nucleotide polymorphisms (SNPs), who had ischemic stroke and underwent pharmacogenomics (PGx) sequencing test. They were recruited from the First Hospital of Jilin University. The inclusion criteria are patients aged above 18 years, with diagnosed stroke, and who underwent pharmacogenomics (PGx) sequencing test, clinical characteristics test, and serum folic acid, B12, and Hcy concentration tests.

The sub-study, including 207 ischemic stroke (177 patients from the first study group and only 30 underwent SLCO1B1c.521T>C polymorphism test) patients and 244 non-ischemic stroke patients who only underwent SLCO1B1c.521T>C polymorphism test to further demonstrate the role of SLCO1B1c.521T>C polymorphism and homocysteine. A total of 30 ischemic stroke patients and 245 non-stroke patients were recruited from April 2021 to October 2024.

### DNA collection and genotyping

Genomic DNA was extracted from 200 µL of whole blood. A total of 55 genes with 99 SNPs were genotyped by the Safe Medication Genetic Test Kit (Semiconductor Sequencing Method, Boao, Beijing) on BioelectronSeq 4000 sequencers. The gene and SNP information are shown in **Supplementary Table S1**. The sub-study patients only underwent the SLCO1B1c.521T>C polymorphism and homocysteine concentration tests. The sub-study samples were genotyped with the SNP assay (GuangYin, Jinan, China) according to the recommended protocol and analyzed with the PCR amplification analysis system (Tianlong, Xian, China).

### Detection of Hcy

Homocysteine (Hcy) levels were measured using EDTA as an anticoagulant to promptly separate plasma from venous blood. The plasma samples were analyzed using a cyclic enzyme assay on a Beckman automated biochemical analyzer at the laboratory of the First Hospital of Jilin University. All Hcy detections were conducted in accordance with the manufacturer’s protocols. For the initial phase of this study, a concentration of 17 μmol/L was designated as the threshold for the high Hcy concentration group. In the subsequent sub-study, the threshold for the high concentration group was set at 15 μmol/L.

### Statistical analysis

We conducted the statistical analysis using SPSS 24.0 for Windows. Quantitative data were described as mean ± standard deviation (mean ± SD), and differences between groups were tested by t-test or one-way ANOVA. We used the median (25% and 75% interquartile range) to describe skewed distribution variables. Odds ratios (ORs) and their 95% confidential intervals (CIs) were calculated as a measure of difference in response rate using logistic regression analysis.

## Result

### Characteristics of the study population

In this study, we included 177 patients diagnosed with ischemic stroke, comprising 141 men and 36 women. The mean age of the male patients was 59.42 ± 11.43 years, whereas for female participants, it was 63.14 ± 11.81 years. The median plasma Hcy, ALT, GGT, TBIL, DBIL, IBIL, uric acid concentrations were significantly higher in men (P = 0.010, P = 0.016, P = 0.003, P = 0.006, P = 0.003, P = 0.009, P < 0.001, respectively).Conversely, the GLB and folic acid concentrations were higher in women (P < 0.001 for both; **Table 1**).

**Table 1:** Characteristics of the patients underwent stroke.

Characteristics	Male(n=141)	Female(n=36)	P
Age (years)	59.42±11.43	63.14±11.81	0.085
AST, U/L	24.55±20.87	21.00±9.16	0.322
ALT ,U/L	26.54±17.91	18.90±11.22	0.016
ALP, U/L	81.97±26.94	89.40±33.41	0.168
GGT, U/L	49.46±53.03	22.21±8.00	0.003
TP, g/L	65.08±5.59	67.49±7.47	0.034
ALB, g/L	38.59±4.10	38.47±3.72	0.871
GLB, g/L	26.47±3.46	29.06±4.97	<0.001
TBIL, umol/L	15.68±7.61	11.95±3.38	0.006
DBIL, umol/L	3.10±1.77	2.19±0.81	0.003
IBIL, umol/L	12.55±6.16	9.76±2.64	0.009
TBA, umol/L	4.12±3.18	3.41±2.21	0.210
ChE, U/L	7649.02±1782.86	7546.38±1651.81	0.755
Uric acid	358.39±98.84	290.54±80.58	<0.001
TC, mmol/L	1.62±1.07	1.68±0.89	0.764
TG, mmol/L	4.24±1.21	4.55±1.02	0.156
HDL, mmol/L	1.02±0.25	1.14±0.27	0.017
LDL, mmol/L	2.69±0.88	2.83±0.72	0.364
Glucose, mmol/L	6.49±2.18	6.81±1.99	0.423
B12, pmol/L	332.22±176.49	337.42±148.75	0.872
Folic acid, nmol/L	7.03±3.62	9.69±3.92	<0.001
HCY, μmol/L	16.14±10.47	11.44±4.22	0.010

AST, Aspartate Aminotransferase; ALT, Alanine Aminotransferase; ALP, Alkaline Phosphatase; GGT, Gamma-Glutamyl Transferase; TP, g/L, Total Protein; ALB, Albumin; GLB, Globulin; TBIL, Total Bilirubin; DBIL, Direct Bilirubin; IBIL, Indirect Bilirubin; TBA, Total Bile Acids; ChE, Cholinesterase; TC, Total Cholesterol; TG, Triglycerides; HDL, High-Density Lipoprotein; LDL, Low-Density Lipoprotein; B12, Vitamin B12; HCY, Homocysteine.

Furthermore, the patients were stratified based on 55 genes and 99 SNPs. The basic information and distribution of the SNPs are summarized in **Supplementary Table S1**.

### Association of 99 SNPs with serum Hcy concentrations

We examined the associations between each genotype and Hcy concentrations and presented only the results that were statistically significant in **Supplementary Table S2**. The highest concentrations of Hcy were demonstrated in patients with the SLCO1B1 521 CC genotype compared with the CT and TT genotypes (P = 0.001). The LTA4H c.-1400 CC and UGT1A1, c.-364 TT also had higher Hcy concentrations (P = 0.025, P = 0.030). There was no significant gene polymorphism difference in other SNPs.

### Univariate and multivariate regression analyses of biochemical factors, serum folate, vitamin B12 concentrations, and SNPs in relation to Hcy concentrations

Logistic regression analysis was performed on the clinical characteristic, serum folate, vitamin B12 concentrations, and SNPs with Hcy concentrations. For men, GGT ≥ 50 U/L, vitamin B12 < 200 pmol/L, folic acid < 4.2 nmol/L, and carries of SLCO1B1 521CC CT were significant risk factors with Hcy concentrations. In the multivariate regression analyses, only vitamin B12 < 200 pmol/L, folic acid < 4.2 nmol/L, and patients carrying SLCO1B1 521CC and CT genotypes were the dependent risk factors, respectively [OR = 3.560 (1.111, 11.405), P = 0.033; OR = 15.433 (5.093, 46.768), P<0.001; OR=3.265 (1.181, 9.028), P=0.023; **Supplementary Table S3**)].

### Univariate and multivariate regression analyses of biochemical factors and SLCO1B1 c.521T>C in relation to Hcy concentrations in ischemic stroke and non-ischemic stroke patients

A total of 207 ischemic stroke patients group (177 patients from the first study patients and only 30 underwent SLCO1B1 c.521T>C polymorphism test) and 244 non-ischemic stroke patients who only underwent SLCO1B1 c.521T>C polymorphism test were included to analyze the SLCO1B1 c.521T>C polymorphism in relation to Hcy concentrations (**Table 2**). Univariate and multivariate regression analyses both showed that being male and SLCO1B1 c.521 CC+CT were independent risk factors for high Hcy concentrations [P < 0.001, OR = 3.347 (1.823, 6.143); P = 0.010, OR = 1.762 (1.102, 2.825), respectively].

**Table 2:** Estimated effects of polymorphisms and other characteristics in logistic regression analyses of Hcy concentrations in ischemic stroke and non-stroke patients.

Group	Characteristics		OR	P	OR	P
All (n = 451)	Sex	Female	3.347 (1.823, 6.143)	<0.001	3.259 (1.771, 5.997)	<0.001
		Male				
	Age	<65 years	1.138 (0.765, 1.695)	0.524		
		≥65 years				
	SLCO1B1, c.521T>C	TT	1.842 (1.160, 2.923)	0.010	1.762 (1.102, 2.825)	0.018
		CC + CT				
Male (n = 355)	Age	<65 years	1.124 (0.727, 1.736)	0.600		
		≥65 years				
	SLCO1B1, c.521T>C	TT	1.814 (1.098, 2.996)	0.020		
		CC + CT				
Female (n = 96)	Age	<65 years	1.845 (0.569, 5.985)	0.308		
		≥65 years				
	SLCO1B1, c.521T>C	TT	1.427 (0.349, 5.830)	0.621		
		CC + CT				

## Discussion

Homocysteine (Hcy), a sulfur-containing amino acid that is not directly obtained from the diet, arises as an intermediate product during the metabolism of methionine. Methionine itself comes from protein-rich foods including poultry, meat, eggs, fish, and dairy. Disruptions in the metabolic pathways of Hcy; insufficient intake of dietary methionine, folate, and vitamins B12, B6, and B2; or genetic factors such as defects, polymorphisms, or mutations affecting enzymes involved in Hcy metabolism, can all contribute to elevated levels of Hcy in the plasma (11). In our retrospective study, the mean Hcy concentrations of ischemic stroke in male patients is 16.14 ± 10.4 μmol/L, higher than in female patients (11.44 ± 4.22 μmol/L). We observed a negative correlation between plasma Hcy concentrations and folic acid and vitamin B12 concentrations, consistent with other studies (12). Research has highlighted elevated homocysteine levels, known as hyper Hcy, as a potential risk factor implicated in a various health conditions, including those affecting the vascular system, neurodegeneration, and ocular health (13). Patients with higher Hcy exhibited impaired glucose metabolism, an altered lipid profile, reduced levels of Vitamin D, and an elevated cardiovascular risk (14). Higher Hcy concentrations are associated with a higher risk of stroke, cardiovascular disease, and Alzheimer’s disease (15, 16). In univariate and multivariate regression analyses, the results demonstrated that low concentrations of folic acid and vitamin B12 can lead to increased plasma concentrations of Hcy.

In the study of the relationship between genotypes and Hcy, we found that Hcy concentrations were significantly higher in the LTA4H rs17525495 c.-1400CC genotype than in the other CT and TT genotypes. Previous studies found that LTA4H rs17525495 was associated with asthma medication montelukast (17), but in univariate regression analysis, LTA4H was not associated with Hcy concentrations.

Similarly, UGT1A1 TT plasma Hcy concentrations were significantly higher than the other CC and CT genotypes, but not significantly different in regression analysis.

Plasma Hcy concentrations were significantly higher in the SLCO1B1 521 CC genotype than in the CT and TT genotypes. In univariate and multivariate regression analyses, SLCO1B1 521 CC and CT genotypes were independent risk factors for elevated plasma Hcy compared to TT genotypes. This finding is the first time the SLCO1B1 gene has been associated with increased plasma Hcy concentrations. The SLCO1B1 gene is located on chromosome 12p12.1 and encodes the organic anion transport polypeptide 1B1 (OATP1B1) protein, an influx transmembrane protein transporter. Apparently, many different influx transporters are responsible for the uptake of various drugs and organic compounds from the bloodstream into the cells. Among these, organic anion-transporting polypeptides (OATPs) are expressed in many organs, including the gut, liver, and kidneys (18). OATPs are critical for understanding the pharmacokinetics of many drugs, including drug absorption, distribution, and elimination. Genetic variations at the SLCO1B1 gene locus can influence drug transport, potentially altering the pharmacokinetic properties of several commonly prescribed medications. Notably, variations in OATP1B1 function are particularly significant for the hepatic uptake of statins and the risk of statin-associated musculoskeletal symptoms (SAMS) (19). Changes in OATP transporter function have been shown to affect the efficacy and safety of many drugs, resulting in instability in drug disposition and response. The Clinical Pharmacology Implementation Consortium (CPIC) and the Dutch Pharmacology Working Group (DPWG) of the Royal Netherlands Society for Pharmaceutical Progress have shown that individuals with decreased function of the organic anion-transporting polypeptide 1B1 (OATP1B1) liver transporter enzyme (encoded by the SLCO1B1 gene) are at increased risk of rhabdomyolysis (20). Our study revealed that the SLCO1B1 polymorphism is associated with serum Hcy concentrations, potentially through its influence on the OATP1B1 liver transporter enzyme. This finding also introduces a novel biomarker for predicting elevated Hcy levels in patients. In patients with diabetes, elevated Hcy levels are associated with an increased risk of diabetic nephropathy in a non-linear fashion (21).

The subgroup analysis revealed that the male sex and SLCO1B1 gene polymorphisms were associated with elevated Hcy concentrations. Specifically, this association was significant in the male group, where SLCO1B1 gene polymorphisms had a pronounced effect. In contrast, among the female group, SLCO1B1 gene polymorphisms were not significantly associated with Hcy levels. This combined validation study also demonstrated that the SLCO1B1 genotype is more significant in male patients. Previous research examines how SLCO1B1 genetic variations affect the pharmacokinetics of statins differently in men and women. The findings indicate that specific SLCO1B1 polymorphisms have a greater impact on statin metabolism in men, potentially leading to higher plasma concentrations and increased risk of adverse effects (22).

The limitation of this study is that it is a retrospective study and there is no pre-study design. The 177 patients with ischemic stroke included at the beginning of the study were small. In order to prove the study conclusion, we then selected 30 ischemic stroke patients and 244 non-stroke patients to prove the relationship between SLCO1B1 gene polymorphism and Hcy, which further confirmed our study conclusion. However, although some of the newly included patients were non-ischemic stroke patients, they were also hospital patients with coronary heart disease rather than healthy controls, so this study could not prove the relationship between SLCO1B1 gene polymorphism and ischemic stroke. Increased clinical studies and mechanism study are necessary to obtain comprehensive profiles of SLCO1B1 gene polymorphism and Hcy. This will allow researchers to better understand molecular mechanisms and how SLCO1B1 gene alters Hcy concentrations. Our study is more significant in the male group, and more mechanism studies are needed to prove the results in the male group.

## Conclusion

Folic acid and vitamin B12 can reduce Hcy concentrations, and SLCO1B1 CT and CC genotypes can lead to elevated Hcy concentrations, especially in the male group.

## Data Availability

The datasets presented in this study can be found in online repositories. The names of the repository/repositories and accession number(s) can be found in the article/**Supplementary Material**.
