# Supplementary material for: The impact of SLCO1B1 polymorphisms on homocysteine concentrations: evidence for a stronger association in men
Source: Front Nephrol. 2025 Jan 29;4:1465380. doi: 10.3389/fneph.2024.1465380 (PMC11815283; doi:10.3389/fneph.2024.1465380)
Supplement: Supplementary file 1 [file Table1.docx]

Supplementary Table 1. Frequency of genotype distribution of polymorphisms in 52 genes

| SNP | Genotype | | Frequency(%) |
| --- | --- | --- | --- |
| ABCB1 | c.2677T>A/G | A/A | 3.38 |
|  |  | A/G | 8.47 |
|  |  | A/T | 17.51 |
|  |  | G/G | 20.34 |
|  |  | G/T | 30.51 |
|  |  | T/T | 19.77 |
| ABCB1 | c.3435T>C | C/C | 35.03 |
|  |  | C/T | 48.02 |
|  |  | T/T | 16.95 |
| ACE | D/I polymorphism | I/I | 42.94 |
|  |  | I/D | 44.07 |
|  |  | D/D | 12.99 |
| ADD1 | c.1378G>T/A | G/G | 22.60 |
|  |  | G/T | 52.54 |
|  |  | T/T | 24.86 |
| ADRB1 | c.1165G>C | C/C | 54.80 |
|  |  | C/G | 38.98 |
|  |  | G/G | 6.22 |
| ADRB2 | c.46A>G | A/A | 32.77 |
|  |  | A/G | 43.50 |
|  |  | G/G | 23.73 |
| AGTR1 | c.*86A>C | A/A | 88.70 |
|  |  | A/C | 10.73 |
|  |  | C/C | 0.57 |
| ALDH2 | c.1510G>A | A/A | 3.95 |
|  |  | A/G | 27.12 |
|  |  | G/G | 68.93 |
| ALOX5 | c.432-6550A>G | A/A | 6.78 |
|  |  | A/G | 32.20 |
|  |  | G/G | 61.02 |
| ANKK1 | c.2137G>A | A/A | 20.34 |
|  |  | A/G | 50.85 |
|  |  | G/G | 28.81 |
| APOE | c.388T>C，c.526C>T | E2/E2 | 0.57 |
|  |  | E2/E3 | 17.51 |
|  |  | E3/E3 | 64.97 |
|  |  | E2/E4 | 1.69 |
|  |  | E4/E4 | 1.12 |
|  |  | E3/E4 | 14.12 |
| C11orf65 | c.175-5285G>T | G/G | 38.42 |
|  |  | G/T | 48.02 |
|  |  | T/T | 13.56 |
| CHIA | c.304G>A/C | A/A | 1.69 |
|  |  | A/G | 17.51 |
|  |  | G/G | 80.79 |
| COMT | c.472G>A | A/A | 8.47 |
|  |  | A/G | 37.85 |
|  |  | G/G | 53.67 |
| CRHR1 | c.1107+111C>T | C/C | 100 |
|  |  | C/T | 0 |
|  |  | T/T | 0 |
| CYP1A1 | c.-30+606G>T | G/G | 80.23 |
|  |  | G/T | 16.38 |
|  |  | T/T | 2.82 |
| CYP2B6 | c.516G>T | G/G | 65.54 |
|  |  | G/T | 31.07 |
|  |  | T/T | 3.39 |
| CYP2C19 | c.636G>A,c.681G>A,c.-806C>T | *1/*17 | 2.26 |
|  |  | *1/*1 | 38.42 |
|  |  | *1/*2 | 41.81 |
|  |  | *1/*3 | 6.21 |
|  |  | *2/*17 | 1.13 |
|  |  | *3/*17 | 0.57 |
|  |  | *2/*3 | 1.12 |
|  |  | *2/*2 | 8.47 |
| CYP2C9 | c.430C>T | C/C | 99.44 |
|  |  | C/T | 0.57 |
|  |  | T/T |  |
|  | c.1075A>C | A/A | 93.79 |
|  |  | A/C | 5.65 |
|  |  | C/C | 0.57 |
| CYP2D6 | g.100C>T | C/C | 24.29 |
|  |  | C/T | 37.85 |
|  |  | T/T | 20.34 |
|  |  | -/C | 6.21 |
|  |  | -/T | 10.73 |
|  | g.984A>G | A/A | 81.36 |
|  |  | A/G | 1.69 |
|  |  | G/G | 0 |
|  |  | -/A | 16.38 |
|  |  | -/G | 0.57 |
|  | g.997C>T/G | C/C | 80.23 |
|  |  | C/G | 2.26 |
|  |  | G/G | 0.56 |
|  |  | -/C | 16.38 |
|  |  | -/G | 0.56 |
|  | g.1758G>A/T | A/G | 1.69 |
|  |  | G/G | 81.36 |
|  |  | -/G | 16.38 |
|  |  | -/A | 0.56 |
|  | g.1846G>A | A/G | 2.25 |
|  |  | G/G | 80.79 |
|  |  | -/G | 15.35 |
|  |  | -/A | 1.69 |
|  | g.2850C>T | C/C | 53.11 |
|  |  | C/T | 25.42 |
|  |  | T/T | 4.52 |
|  |  | -/C | 14.12 |
|  |  | -/T | 2.82 |
|  | g.2988G>A | A/G | 6.21 |
|  |  | G/G | 76.84 |
|  |  | -/G | 16.38 |
|  |  | -/A | 0.56 |
|  |  | A/A |  |
|  | g.3384A>C | A/A | 7.34 |
|  |  | A/C | 35.02 |
|  |  | C/C | 40.68 |
|  |  | -/A | 3.95 |
|  |  | -/C | 12.99 |
|  | g.3435C>A | C/C | 83.05 |
|  |  | -/C | 16.95 |
|  |  | C/A | 0 |
|  |  | A/A | 0 |
|  |  | -/A | 0 |
|  | g.4172C>T/G | C/C | 83.05 |
|  |  | -/C | 16.95 |
|  |  | C/T | 0 |
|  |  | C/G | 0 |
|  |  | -/T | 0 |
|  | g.4180G>C | C/C | 40.68 |
|  |  | C/G | 32.77 |
|  |  | G/G | 9.60 |
|  |  | -/C | 13.56 |
|  |  | -/G | 3.39 |
|  | full-gene-deletion | fullGene/fullGene | 83.05 |
|  |  | deletion/fullGene | 16.95 |
| CYP3A4 | c.1026+12G>A | A/A | 3.96 |
|  |  | A/G | 35.03 |
|  |  | G/G | 61.01 |
| CYP3A5 | c.-253-1G>A | A/A | 4.52 |
|  |  | A/G | 37.85 |
|  |  | G/G | 57.63 |
| CYP4F2 | c.1297G>A | A/A | 11.86 |
|  |  | A/G | 38.42 |
|  |  | G/G | 49.72 |
| DRD2 | c.-585A>G | A/A | 69.49 |
|  |  | A/G | 27.12 |
|  |  | G/G | 3.39 |
| EPHX1 | c.337T>C | C/C | 12.99 |
|  |  | C/T | 56.50 |
|  |  | T/T | 30.51 |
|  | c.416A>G | A/A | 80.79 |
|  |  | A/G | 18.64 |
|  |  | G/G | 0.57 |
| LDLR | c.*666T>C | C/C | 54.80 |
|  |  | C/T | 38.29 |
|  |  | T/T | 7.91 |
| LTA4H | c.-1400C>T | C/C | 27.68 |
|  |  | C/T | 50.28 |
|  |  | T/T | 22.04 |
| LTC4S | c.-444A>C | A/A | 76.27 |
|  |  | A/C | 21.47 |
|  |  | C/C | 2.26 |
| MT-RNR1 | m.1494C>T | C/C | 100 |
|  |  | C/T | 0 |
|  |  | T/T | 0 |
|  | m.1555A>G | A/A | 100 |
|  |  | A/G | 0 |
|  |  | G/G | 0 |
| NAT2 | c.282C>T | C/C | 35.03 |
|  |  | C/T | 51.41 |
|  |  | T/T | 13.56 |
|  | c.341T>C | C/C | 0 |
|  |  | C/T | 6.21 |
|  |  | T/T | 93.79 |
|  | c.481C>T | C/C | 93.78 |
|  |  | C/T | 6.22 |
|  |  | T/T | 0 |
|  | c.590G>A | A/A | 2.26 |
|  |  | A/G | 40.68 |
|  |  | G/G | 57.06 |
|  | c.803G>A | A/A | 92.66 |
|  |  | A/G | 7.34 |
|  |  | G/G | 0 |
|  | c.857G>A | A/A | 2.26 |
|  |  | A/G | 28.81 |
|  |  | G/G | 68.93 |
| NOS1AP | c.178-13122C>T | C/C | 35.59 |
|  |  | C/T | 44.07 |
|  |  | T/T | 20.34 |
| NUDT15 | c.52G>A | A/A | 0 |
|  |  | A/G | 2.26 |
|  |  | G/G | 97.74 |
|  | c.55_56insGAGTCG | GAGTCG/GAGTCG | 0.56 |
|  |  | -/GAGTCG | 11.30 |
|  |  | -/- | 88.14 |
|  | c.415C>T | C/C | 78.53 |
|  |  | C/T | 20.34 |
|  |  | T/T | 1.13 |
|  | c.416G>A | G/G | 99.44 |
|  |  | A/G | 0.56 |
|  |  | A/A | 0 |
| OPRM1 | c.118A>G | A/G | 44.07 |
|  |  | A/A | 49.72 |
|  |  | G/G | 6.21 |
| POLG | c.1399G>A | G/G | 100 |
|  |  | G/A |  |
|  |  | A/A |  |
| PPARG | c.34C>G | C/C | 88.70 |
|  |  | C/G | 11.30 |
|  |  | G/G | 0 |
| SCN1A | c.603-91G>A | A/A | 19.77 |
|  |  | A/G | 52.54 |
|  |  | G/G | 27.68 |
| SCN2A | c.56G>A | A/A | 1.69 |
|  |  | A/G | 22.60 |
|  |  | G/G | 75.71 |
|  | c.971-32A>G | A/A | 84.75 |
|  |  | A/G | 14.69 |
|  |  | G/G | 0.56 |
| SLC22A1 | c.1222A>C/G | A/A | 5.65 |
|  |  | A/G | 43.50 |
|  |  | G/G | 50.85 |
| SLC22A2 | c.808T>G | T/T | 2.27 |
|  |  | G/T | 17.51 |
|  |  | G/G | 80.22 |
| SLC47A1 | c.922-158G>A | A/A | 22.03 |
|  |  | A/G | 51.41 |
|  |  | G/G | 25.96 |
| SLCO1B1 | c.521T>C | C/C | 1.69 |
|  |  | C/T | 18.64 |
|  |  | T/T | 79.66 |
| STXBP1 | c.922A>T | A/A | 100 |
|  |  | A/T | 0 |
|  |  | T/T | 0 |
| TPMT | c.719A>G/C | A/A | 95.48 |
|  |  | A/G | 3.96 |
|  |  | G/G | 0.56 |
| UGT1A | c.*211T>C | C/C | 72.32 |
|  |  | C/T | 27.12 |
|  |  | T/T | 0.56 |
|  | c.*339G>C | C/C | 72.32 |
|  |  | C/G | 27.12 |
|  |  | G/G | 0.56 |
| UGT1A1 | c.211G>A | A/A | 4.52 |
|  |  | A/G | 37.29 |
|  |  | G/G | 58,19 |
|  | c.-364C>T | C/C | 74.58 |
|  |  | C/T | 23.73 |
|  |  | T/T | 1.69 |
| UGT1A4 | c.142T>G/A | G/G | 6.22 |
|  |  | G/T | 24.29 |
|  |  | T/T | 69.49 |
| UGT2B15 | c.253T>G | G/G | 26.55 |
|  |  | G/T | 49.72 |
|  |  | T/T | 23.73 |
| VKORC1 | c.174-136C>T | C/C | 0.56 |
|  |  | C/T | 12.44 |
|  |  | T/T | 87.00 |
|  | c.-1639G>A | A/G | 12.43 |
|  |  | G/G | 0.56 |
|  |  | A/A | 87.00 |
| G6PD | c.95A>G | A/A | 100 |
|  | c.196T>A | T/T | 100 |
|  | c.202G>A | G/G | 100 |
|  | c.392G>T | G/G | 100 |
|  | c.487G>A | G/G | 100 |
|  | c.493A>G | A/A | 100 |
|  | c.517T>C | T/T | 100 |
|  | c.519C>T | C/C | 100 |
|  | c.563C>T | C/C | 100 |
|  | c.592C>T | C/C | 100 |
|  | c.871G>A | G/G | 100 |
|  | c.1004C>T | C/C | 100 |
|  | c.1024C>T | C/C | 100 |
|  | c.1360C>T | C/C | 100 |
|  | c.1376G>T | G/G | 100 |
|  | c.1388G>A | G/G | 100 |
| GRIK4 | c.83-10039T>C | C/C | 65.54 |
|  |  | C/T | 28.81 |
|  |  | T/T | 5.65 |
| HLA-A | *3101 | positive | 9.60 |
|  |  | negative | 90.40 |
| HLA-B | *1502 | positive | 5.08 |
|  |  | negative | 94.92 |
|  | *5801 | positive | 7.34 |
|  |  | negative | 92.66 |
| HTR1A | c.-1019G>C | C/C | 56.50 |
|  |  | C/G | 35.03 |
|  |  | G/G | 8.47 |
| IFNL4 | g.1332A>C | A/A | 91.53 |
|  |  | A/C | 7.34 |
|  |  | C/C | 1.13 |
|  | g.5710G>A | A/A | 1.13 |
|  |  | A/G | 7.91 |
|  |  | G/G | 90.96 |
| ITPA | c.94C>A/G | A/A | 2.82 |
|  |  | A/C | 19.77 |
|  |  | C/C | 77.40 |
|  | c.124+21A>C | A/A | 99.44 |
|  |  | A/C | 0.56 |
|  |  | C/C | 0 |
