# Supplementary material for: The impact of SLCO1B1 polymorphisms on homocysteine concentrations: evidence for a stronger association in men
Source: Front Nephrol. 2025 Jan 29;4:1465380. doi: 10.3389/fneph.2024.1465380 (PMC11815283; doi:10.3389/fneph.2024.1465380)
Supplement: Supplementary file 2 [file Table2.docx]

Table 2 Relationship between SNPs and Hcy levels in patient with Stroke.

|  |  |  | HCY |  |
| --- | --- | --- | --- | --- |
| GENE | SNP |  | ALL | P |
| ABCB1 | c.2677T>A/G | A/A | 20.13±7.58 | 0.190 |
|  |  | A/G | 16.19±12.92 |  |
|  |  | A/T | 15.43±9.71 |  |
|  |  | G/G | 17.94±13.94 |  |
|  |  | G/T | 12.91±4.86 |  |
|  |  | T/T | 14.43±8.14 |  |
| ABCB1 | c.3435T>C | C/C | 16.97±12.72 | 0.177 |
|  |  | C/T | 13.91±7.00 |  |
|  |  | T/T | 15.06±8.82 |  |
| ACE | D/I polymorphism | I/I | 16.98±12.72 | 0.216 |
|  |  | I/D | 13.91±7.00 |  |
|  |  | D/D | 15.06±8.82 |  |
| ADD1 | c.1378G>T/A | G/G | 13.80±7.68 | 0.436 |
|  |  | G/T | 16.60±12.12 |  |
|  |  | T/T | 15.30±6.27 |  |
| ADRB1 | c.1165G>C | C/C | 13.70±4.78 | 0.186 |
|  |  | C/G | 16.02±11.34 |  |
|  |  | G/G | 14.75±9.27 |  |
| ADRB2 | c.46A>G | A/A | 14.07±7.51 | 0.331 |
|  |  | A/G | 16.85±12.27 |  |
|  |  | G/G | 14.17±7.10 |  |
| AGTR1 | c.*86A>C | A/A | 13.69±6.72 | 0.823 |
|  |  | A/C | 16.22±10.87 |  |
|  |  | C/C | 15.45±10.99 |  |
| ALDH2 | c.1510G>A | A/A | 11.56±4.61 | 0.458 |
|  |  | A/G | 16.26±11.15 |  |
|  |  | G/G | 14.99±9.34 |  |
| ALOX5 | c.432-6550A>G | A/A | 21.15±18.96 | 0.070 |
|  |  | A/G | 15.78±10.56 |  |
|  |  | G/G | 14.26±7.57 |  |
| ANKK1 | c.2137G>A | A/A | 13.10±4.73 | 0.817 |
|  |  | A/G | 15.85±11.16 |  |
|  |  | G/G | 14.55±6.53 |  |
| APOE | c.388T>C，c.526C>T | E2/E2 | 15.93±12.04 | 0.386 |
|  |  | E2/E3 |  |  |
|  |  | E3/E3 | 15.23±8.85 |  |
|  |  | E2/E4 |  |  |
|  |  | E4/E4 | 14.58±9.48 |  |
|  |  | E3/E4 |  |  |
| C11orf65 | c.175-5285G>T | G/G | 14.58±9.48 | 0.512 |
|  |  | G/T | 15.23±8.85 |  |
|  |  | T/T | 15.93±12.04 |  |
| CHIA | c.304G>A/C | A/A | 15.94±11.82 | 0.630 |
|  |  | A/G | 15.17±9.14 |  |
|  |  | G/G | 13.24±3.37 |  |
| COMT | c.472G>A | A/A | 20.45±3.88 | 0.627 |
|  |  | A/G | 15.43±13.65 |  |
|  |  | G/G | 15.03±8.83 |  |
| CRHR1 | c.1107+111C>T | C/C | / | / |
|  |  | C/T |  |  |
|  |  | T/T |  |  |
| CYP1A1 | c.-30+606G>T | G/G | 16.81±15.52 | 0.938 |
|  |  | G/T | 15.67±9.91 |  |
|  |  | T/T | 14.58±8.39 |  |
| CYP2B6 | c.516G>T | G/G | 15.24±10.07 | 0.796 |
|  |  | G/T | 15.21±8.8 |  |
|  |  | T/T | 17.72±14.16 |  |
| CYP2C19 | c.636G>A,c.681G>A,c.-806C>T | *1/*17 | 13.88±8.93 | 0.337 |
|  |  | *1/*1 |  |  |
|  |  | *1/*2 | 16.19±10.39 |  |
|  |  | *1/*3 |  |  |
|  |  | *2/*17 |  |  |
|  |  | *3/*17 |  |  |
|  |  | *2/*3 | 15.50±9.22 |  |
|  |  | *2/*2 |  |  |
| CYP2C9 | c.430C>T | C/C | 15.21±9.74 | 0.796 |
|  |  | C/T | 12.68 |  |
|  |  | T/T | / |  |
|  | c.1075A>C | A/A | 15.15±9.94 | 0.767 |
|  |  | A/C | 16.37±5.78 |  |
|  |  | C/C | 9.14 |  |
| CYP2D6 | g.100C>T | C/C | 14.19±8.89 | 0.854 |
|  |  | C/T | 15.81±12.05 |  |
|  |  | T/T | 15.18±7.29 |  |
|  |  | -/C |  |  |
|  |  | -/T |  |  |
|  | g.984A>G | A/A | 15.19±10.21 | 0.999 |
|  |  | A/G | 14.41±2.14 |  |
|  |  | G/G | / |  |
|  |  | -/A | 15.25±7.80 |  |
|  |  | -/G | 15.54 |  |
|  | g.997C>T/G | C/C | 15.26±10.26 | 0.961 |
|  |  | C/G | 12.63±3.95 |  |
|  |  | G/G | 14.06 |  |
|  |  | -/C | 15.24±7.80 |  |
|  |  | -/G | 15.54 |  |
|  | g.1758G>A/T | A/G | 9.38±0.23 | 0.616 |
|  |  | G/G | 15.30±10.18 |  |
|  |  | -/G | 15.54±7.63 |  |
|  |  | -/A | 7.16 |  |
|  | g.1846G>A | A/G | 13.35±2.74 | 0.899 |
|  |  | G/G | 15.23±10.24 |  |
|  |  | -/G | 15.67±7.94 |  |
|  |  | -/A | 11.70±3.42 |  |
|  | g.2850C>T | C/C | 16.12±11.37 | 0.489 |
|  |  | C/T | 13.77±7.49 |  |
|  |  | T/T | 11.79±3.67 |  |
|  |  | -/C | 15.95±7.89 |  |
|  |  | -/T | 11.92±5.94 |  |
|  | g.2988G>A | A/G | 13.53±4.31 | 0.843 |
|  |  | G/G | 15.31±10.44 |  |
|  |  | -/G | 15.50±7.69 |  |
|  |  | -/A | 8.53 |  |
|  |  | A/A | / |  |
|  | g.3384A>C | A/A | 16.73±13.47 | 0.831 |
|  |  | A/C | 15.93±11.98 |  |
|  |  | C/C | 14.23±7.31 |  |
|  |  | -/A | 14.01±3.30 |  |
|  |  | -/C | 15.58±8.46 |  |
|  | g.3435C>A | C/C | 15.18±10.11 | 0.968 |
|  |  | -/C | 15.26±7.66 |  |
|  |  | C/A | / |  |
|  |  | A/A | / |  |
|  |  | -/A | / |  |
|  | g.4172C>T/G | C/C | 15.18±10.11 | 0.968 |
|  |  | -/C | 15.26±7.66 |  |
|  |  | C/T |  |  |
|  |  | C/G |  |  |
|  |  | -/T |  |  |
|  | g.4180G>C | C/C | 14.25±7.30 | 0.837 |
|  |  | C/G | 16.32±12.29 |  |
|  |  | G/G | 15.11±12.09 |  |
|  |  | -/C |  |  |
|  |  | -/G |  |  |
|  | full-gene-deletion | fullGene/fullGene | 15.17±10.10 | 0.968 |
|  |  | deletion/fullGene | 15.25±7.66 |  |
| CYP3A4 | c.1026+12G>A | A/A | 17.78±9.29 | 0.726 |
|  |  | A/G | 14.72±9.20 |  |
|  |  | G/G | 15.29±10.09 |  |
| CYP3A5 | c.-253-1G>A | A/A | 11.98±5.83 | 0.635 |
|  |  | A/G | 15.32±9.01 |  |
|  |  | G/G | 15.36±10.43 |  |
| CYP4F2 | c.1297G>A | A/A | 14.30±5.59 | 0.429 |
|  |  | A/G | 16.41±11.56 |  |
|  |  | G/G | 14.45±8.86 |  |
| DRD2 | c.-585A>G | A/A | 15.19±9.83 | 0.992 |
|  |  | A/G | 15.25±10.03 |  |
|  |  | G/G | 14.70±4.57 |  |
| EPHX1 | c.337T>C | C/C | 18.64±17.09 | 0.203 |
|  |  | C/T | 14.75±9.12 |  |
|  |  | T/T | 14.57±5.79 |  |
|  | c.416A>G | A/A | 15.50±10.51 | 0.683 |
|  |  | A/G | 13.96±5.46 |  |
|  |  | G/G | 12.08 |  |
| LDLR | c.*666T>C | C/C | 14.98±10.53 | 0.831 |
|  |  | C/T | 15.70±9.19 |  |
|  |  | T/T | 14.14±5.67 |  |
| LTA4H | c.-1400C>T | C/C | 18.37±14.00 | 0.025 |
|  |  | C/T | 14.37±7.43 |  |
|  |  | T/T | 13.16±6.84 |  |
| LTC4S | c.-444A>C | A/A | 14.59±8.05 | 0.170 |
|  |  | A/C | 17.78±14.43 |  |
|  |  | C/C | 11.84±4.83 |  |
| MT-RNR1 | m.1494C>T | C/C | / |  |
|  |  | C/T | / |  |
|  |  | T/T | / |  |
|  | m.1555A>G | A/A | / |  |
|  |  | A/G | / |  |
|  |  | G/G | / |  |
| NAT2 | c.282C>T | C/C | 15.17±9.92 | 0.925 |
|  |  | C/T | 15.38±10.18 |  |
|  |  | T/T | 14.48±7.40 |  |
|  | c.341T>C | C/C | / | 0.376 |
|  |  | C/T | 12.67±5.04 |  |
|  |  | T/T | 15.36±9.94 |  |
|  | c.481C>T | C/C | 15.36±9.94 | 0.376 |
|  |  | C/T | 12.67±5.04 |  |
|  |  | T/T |  |  |
|  | c.590G>A | A/A | 11.09±6.28 | 0.637 |
|  |  | A/G | 15.80±10.38 |  |
|  |  | G/G | 14.88±9.34 |  |
|  | c.803G>A | A/A | 15.04±9.05 | 0.477 |
|  |  | A/G | 17.04±16.27 |  |
|  |  | G/G |  |  |
|  | c.857G>A | A/A | 15.83±10.55 | 0.804 |
|  |  | A/G | 14.43±7.92 |  |
|  |  | G/G | 15.49±10.44 |  |
| NOS1AP | c.178-13122C>T | C/C | 14.94±8.95 | 0.864 |
|  |  | C/T | 15.02±9.52 |  |
|  |  | T/T | 15.97±11.49 |  |
| NUDT15 | c.52G>A | A/A | / | 0.922 |
|  |  | A/G | 15.66±5.06 |  |
|  |  | G/G | 15.18±9.81 |  |
|  | c.55_56insGAGTCG | GAGTCG/GAGTCG | 22.95 | 0.438 |
|  |  | -/GAGTCG | 13.17±4.58 |  |
|  |  | -/- | 15.41±10.20 |  |
|  | c.415C>T | C/C | 15.24±10.55 | 0.811 |
|  |  | C/T | 15.26±5.89 |  |
|  |  | T/T | 10.77±2.91 |  |
|  | c.416G>A | G/G | 15.11±9.68 | 0.121 |
|  |  | A/G | 30.22 |  |
|  |  | A/A | / |  |
| OPRM1 | c.118A>G | A/G | 15.35±10.83 | 0.881 |
|  |  | A/A | 14.89±8.91 |  |
|  |  | G/G | 16.35±7.72 |  |
| POLG | c.1399G>A | G/G | / | / |
|  |  | G/A | / |  |
|  |  | A/A | / |  |
| PPARG | c.34C>G | C/C | 15.41±10.11 | 0.422 |
|  |  | C/G | 13.54±5.81 |  |
|  |  | G/G |  |  |
| SCN1A | c.603-91G>A | A/A | 15.31±8.18 | 0.723 |
|  |  | A/G | 15.65±10.53 |  |
|  |  | G/G | 14.26±9.25 |  |
| SCN2A | c.56G>A | A/A | 19.41±5.21 | 0.624 |
|  |  | A/G | 15.95±13.24 |  |
|  |  | G/G | 14.86±8.52 |  |
|  | c.971-32A>G | A/A | 15.42±9.99 | 0.454 |
|  |  | A/G | 13.58±8.05 |  |
|  |  | G/G | 21.07 |  |
| SLC22A1 | c.1222A>C/G | A/A | 17.62±19.51 | 0.460 |
|  |  | A/G | 15.84±10.87 |  |
|  |  | G/G | 14.38±6.78 |  |
| SLC22A2 | c.808T>G | T/T | 12.06±2.40 | 0.704 |
|  |  | G/T | 14.41±9.46 |  |
|  |  | G/G | 15.45±9.92 |  |
| SLC47A1 | c.922-158G>A | A/A | 15.03±10.11 | 0.066 |
|  |  | A/G | 13.88±7.34 |  |
|  |  | G/G | 18.05±12.85 |  |
| SLCO1B1 | c.521T>C | C/C | 37.88±26.99 | 0.000 |
|  |  | C/T | 16.75±11.57 |  |
|  |  | T/T | 14.33±8.07 |  |
| STXBP1 | c.922A>T | A/A | / | / |
|  |  | A/T |  |  |
|  |  | T/T |  |  |
| TPMT | c.719A>G/C | A/A | 15.27±9.92 | 0.855 |
|  |  | A/G | 13.98±3.79 |  |
|  |  | G/G | 10.88 |  |
| UGT1A | c.*211T>C | C/C | 15.75±10.34 | 0.427 |
|  |  | C/T | 13.84±7.84 |  |
|  |  | T/T | 9.10 |  |
|  | c.*339G>C | C/C | 15.75±10.34 | 0.427 |
|  |  | C/G | 13.84±7.84 |  |
|  |  | G/G | 9.1 |  |
| UGT1A1 | c.-53_-52TA | TA/TA | / | / |
|  | c.211G>A | A/A | 17.01±7.73 | 0.802 |
|  |  | A/G | 14.74±9.92 |  |
|  |  | G/G | 15.34±9.79 |  |
|  | c.-364C>T | C/C | 15.14±10.45 | 0.030 |
|  |  | C/T | 14.29±5.11 |  |
|  |  | T/T | 29.61±16.79 |  |
| UGT1A4 | c.142T>G/A | G/G | 11.92±3.74 | 0.386 |
|  |  | G/T | 14.42±8.31 |  |
|  |  | T/T | 15.76±10.49 |  |
| UGT2B15 | c.253T>G | G/G | 13.20±8.42 | 0.119 |
|  |  | G/T | 15.14±7.17 |  |
|  |  | T/T | 17.48±14.27 |  |
| VKORC1 | c.174-136C>T | C/C | 12.83 | 0.938 |
|  |  | C/T | 14.69±7.74 |  |
|  |  | T/T | 15.28±10.02 |  |
|  | c.-1639G>A | A/G | 14.69±7.74 | 0.938 |
|  |  | G/G | 12.83 |  |
|  |  | A/A | 15.28±10.02 |  |
| G6PD | c.95A>G | A/A |  | / |
|  | c.196T>A | T/T |  | / |
|  | c.202G>A | G/G |  | / |
|  | c.392G>T | G/G |  | / |
|  | c.487G>A | G/G |  | / |
|  | c.493A>G | A/A |  | / |
|  | c.517T>C | T/T |  | / |
|  | c.519C>T | C/C |  | / |
|  | c.563C>T | C/C |  | / |
|  | c.592C>T | C/C |  | / |
|  | c.871G>A | G/G |  | / |
|  | c.1004C>T | C/C |  | / |
|  | c.1024C>T | C/C |  | / |
|  | c.1360C>T | C/C |  | / |
|  | c.1376G>T | G/G |  | / |
|  | c.1388G>A | G/G |  | / |
| GRIK4 | c.83-10039T>C | C/C | 14.29±7.96 | 0.167 |
|  |  | C/T | 17.44±13,13 |  |
|  |  | T/T | 14.77±8.00 |  |
| HLA-A | *3101 | positive | 16.06±4.84 | 0.697 |
|  |  | negative | 15.09±10.11 |  |
| HLA-B | *1502 | positive | 12.01±2.84 | 0.315 |
|  |  | negative | 15.36±9.93 |  |
|  | *5801 | positive | 13.79±5.47 | 0.592 |
|  |  | negative | 15.31±9.99 |  |
| HTR1A | c.-1019G>C | C/C | 14.43±6.73 | 0.481 |
|  |  | C/G | 16.35±13.00 |  |
|  |  | G/G | 15.35±10.57 |  |
| IFNL4 | g.1332A>C | A/A | 15.39±9.84 | 0.613 |
|  |  | A/C | 13.59±8.93 |  |
|  |  | C/C | 9.99±1.54 |  |
|  | g.5710G>A | A/A | 9.99±1.54 | 0.594 |
|  |  | A/G | 13.54±8.57 |  |
|  |  | G/G | 15.41±9.87 |  |
| ITPA | c.94C>A/G | A/A | 12.77±1.67 | 0.878 |
|  |  | A/C | 15.11±8.92 |  |
|  |  | C/C | 15.29±10.08 |  |
|  | c.124+21A>C | A/A | 15.08±9.65 | 0.067 |
|  |  | A/C | 32.97 |  |
|  |  | C/C | / |  |
