# Supplementary material for: The impact of SLCO1B1 polymorphisms on homocysteine concentrations: evidence for a stronger association in men
Source: Front Nephrol. 2025 Jan 29;4:1465380. doi: 10.3389/fneph.2024.1465380 (PMC11815283; doi:10.3389/fneph.2024.1465380)
Supplement: Supplementary file 3 [file Table3.docx]

Table 3. Estimated effects of polymorphisms in logistic regression analyses of HCY

| Characteristics |  |  | **OR** | **P** | OR | P |
| --- | --- | --- | --- | --- | --- | --- |
| Sex | Female |  | 5.824(1.330, 25.506) | 0.019 | 2.470(0.500,12.209) | 0.267 |
|  | Male |  |  |  |  |  |
| Age | <65, years |  | 1.504(0.719, 3.145) | 0.278 |  |  |
|  | ≥65，years |  |  |  |  |  |
| AST, U/L | <40 |  | 1.684(0.488, 5.810) | 0.410 |  |  |
|  | ≥40 |  |  |  |  |  |
| ALT ,U/L | <40 |  | 1.330(0.514,3.439) | 0.556 |  |  |
|  | ≥40 |  |  |  |  |  |
| ALP, U/L | <130 |  | 1.200(0.232, 6.208) | 0.828 |  |  |
|  | ≥130 |  |  |  |  |  |
| GGT, U/L | <50 |  | 2.482(1.155, 5.334) | 0.020 | 2.216(0.849,5.782) | 0.104 |
|  | ≥50 |  |  |  |  |  |
| TP,g/L | ≥60 |  | 0.426(0.120,1.506) | 0.185 |  |  |
|  | <60 |  |  |  |  |  |
| ALB, g/L | ≥35 |  | 1.535(0.550, 4.284) | 0.413 |  |  |
|  | <35 |  |  |  |  |  |
| GLB, g/L | <30 |  | 0.938(0.388,2.266) | 0.887 |  |  |
|  | ≥30 |  |  |  |  |  |
| TBIL, umol/L | <20 |  | 1.513(0.544, 4.206) | 0.427 |  |  |
|  | ≥20 |  |  |  |  |  |
| DBIL, umol/L | <7 |  | 1.213(0.122, 12.014) | 0.869 |  |  |
|  | ≥7 |  |  |  |  |  |
| IBIL, umol/L | <10 |  | 1.325(0.622, 2.826) | 0.466 |  |  |
|  | ≥10 |  |  |  |  |  |
| TBA, umol/L | <10 |  | 1.029(0.204, 5.174) | 0.973 |  |  |
|  | ≥10 |  |  |  |  |  |
| ChE, U/L | <8000 |  | 1.169(0.562,2.431) | 0.676 |  |  |
|  | ≥8000 |  |  |  |  |  |
| Uric acid | <380 |  | 1.306(0.604, 2.823) | 0.498 |  |  |
|  | ≥380 |  |  |  |  |  |
| TC, mmol/L | <1.7 |  | 0.963(0.451, 2.055) | 0.921 |  |  |
|  | ≥1.7 |  |  |  |  |  |
| TG, mmol/L | <5.20 |  | 1.475(0.654, 3.325) | 0.349 |  |  |
|  | ≥5.20 |  |  |  |  |  |
| HDL, mmol/L | <1.00 |  | 0.698(0.333,1.466) | 0.342 |  |  |
|  | ≥1,00 |  |  |  |  |  |
| LDL, mmol/L | <3.40 |  | 1.124(0.479, 2.638) | 0.789 |  |  |
|  | ≥3.40 |  |  |  |  |  |
| Glucose, mmol/L | <6.11 |  | 0.511(0.234, 1.115) | 0.092 |  |  |
|  | ≥6.11 |  |  |  |  |  |
| B12 | ≥200 |  | 3.178(1.314, 7.688) | 0.010 | 3.560(1.111,11.405) | 0.033 |
|  | <200 |  |  |  |  |  |
| Folic acid | ≥4.2 |  | 17.857(6.623,48.149) | 0.000 | 15.433(5.093,46.768) | <0.001 |
|  | <4.2 |  |  |  |  |  |
| ABCB1 | c.2677T>A/G | A/A |  | 0.086 |  |  |
|  |  | A/G | 9.000(1.189,68.133) | 0.033 |  |  |
|  |  | A/T | 1.500(0.309,7.284) | 0.615 |  |  |
|  |  | G/G | 1,200(0.312,4.622) | 0.791 |  |  |
|  |  | G/T | 3.391(1.057,10.880)) | 0.040 |  |  |
|  |  | T/T | 1.256(0.383,4.122) | 0.707 |  |  |
| ABCB1 | c.3435T>C | C/C |  | 0.212 |  |  |
|  |  | C/T | 0.535(0.244, 1.173) | 0.118 |  |  |
|  |  | T/T | 0.478(0.158, 1.445) | 0.191 |  |  |
| ACE | D/I polymorphism | I/I |  | 0.215 |  |  |
|  |  | I/D | 1.422(0.635,3.187) | 0.392 |  |  |
|  |  | D/D | 2.544(0.894, 7.238) | 0.080 |  |  |
| ADD1 | c.1378G>T/A | G/G |  | 0.303 |  |  |
|  |  | G/T | 2.267(0.793, 6.482) | 0.127 |  |  |
|  |  | T/T | 2,125(0.655, 6.895) | 0.209 |  |  |
| ADRB1 | c.1165G>C | C/C |  | 0.566 |  |  |
|  |  | C/G | 1.471(0.699, 3.094) | 0.310 |  |  |
|  |  | G/G | 0.926(0.184, 4.661) | 0.926 |  |  |
| ADRB2 | c.46A>G | A/A |  | 0.905 |  |  |
|  |  | A/G | 1.057(0.455, 2.456) | 0.897 |  |  |
|  |  | G/G | 1.237(0.476, 3.213) | 0.663 |  |  |
| AGTR1 | c.*86A>C | A/A |  | 0.896 |  |  |
|  |  | A/C | 1.299(0.436, 3.868) | 0.639 |  |  |
|  |  | C/C | 0.000 | 1.000 |  |  |
| ALDH2 | c.1510G>A | A/A |  | 0.896 |  |  |
|  |  | A/G | 1.299(0.436, 3.868) | 0.639 |  |  |
|  |  | G/G | 0.000 | 1.000 |  |  |
| ALOX5 | c.432-6550A>G | A/A |  | 0.468 |  |  |
|  |  | A/G | 1.573(0.731,3.384) | 0.246 |  |  |
|  |  | G/G | 1.612(0.392, 6.627) | 0.508 |  |  |
| ANKK1 | c.2137G>A | A/A |  | 0.484 |  |  |
|  |  | A/G | 1.670(0.678, 4.115) | 0.265 |  |  |
|  |  | G/G | 1.750(0.601, 5.092) | 0.304 |  |  |
| APOE | c.388T>C，c.526C>T | E2/E2 |  | 0.895 |  |  |
|  |  | E2/E3 |  |  |  |  |
|  |  | E3/E3 | 1.002(0.388, 2.586) | 0.997 |  |  |
|  |  | E2/E4 |  |  |  |  |
|  |  | E4/E4 | 0.779(0.216, 2.817) | 0.704 |  |  |
|  |  | E3/E4 |  |  |  |  |
| C11orf65 | c.175-5285G>T | G/G |  | 0.796 |  |  |
|  |  | G/T | 0.947(0.451, 2.106) | 0.947 |  |  |
|  |  | T/T | 0.863(0.197, 2.255) | 0.514 |  |  |
| CHIA | c.304G>A/C | A/A |  | 0.617 |  |  |
|  |  | A/G | 0.000 | 0.999 |  |  |
|  |  | G/G | 0.000 | 0.999 |  |  |
| COMT | c.472G>A | A/A |  | 0.367 |  |  |
|  |  | A/G | 2.437(0.500,11.884) | 0.478 |  |  |
|  |  | G/G | 1.581(0.327,7.640) | 0.899 |  |  |
| CRHR1 | c.1107+111C>T | C/C | / |  |  |  |
|  |  | C/T |  |  |  |  |
|  |  | T/T |  |  |  |  |
| CYP1A1 | c.-30+606G>T | G/G |  | 0.950 |  |  |
|  |  | G/T | 1.156(0.451,2.964) | 0.763 |  |  |
|  |  | T/T | 0.908(0.098,8.433) | 0.933 |  |  |
| CYP2B6 | c.516G>T | G/G |  | 0.883 |  |  |
|  |  | G/T | 0.856(0.378,1.894) | 0.701 |  |  |
|  |  | T/T | 0.669(0.075,5.987) | 0.719 |  |  |
| CYP2C19 | c.636G>A,c.681G>A,c.-806C>T | *1/*17 |  | 0.084 |  |  |
|  |  | *1/*1 |  |  |  |  |
|  |  | *1/*2 | 2.459(1.088,5.557) | 0.031 |  |  |
|  |  | *1/*3 |  |  |  |  |
|  |  | *2/*17 |  |  |  |  |
|  |  | *3/*17 |  |  |  |  |
|  |  | *2/*3 | 1.286(0.312.5.294) | 0.728 |  |  |
|  |  | *2/*2 |  |  |  |  |
| CYP2C9 | c.430C>T | C/C | 0.00 | 1.000 |  |  |
|  |  | C/T |  |  |  |  |
|  |  | T/T |  |  |  |  |
|  | c.1075A>C | A/A |  | 0.987 |  |  |
|  |  | A/C | 0.875(0.178.4.304) | 0.870 |  |  |
|  |  | C/C | 0.000 | 1.000 |  |  |
| CYP2D6 | g.100C>T | C/C |  | 0.869 |  |  |
|  |  | C/T | 1.144(0.434,3.019) | 0.785 |  |  |
|  |  | T/T | 1.593(0.404,3.649) | 0.729 |  |  |
|  |  | -/C | 2.536(0.570,6.755) | 0.286 |  |  |
|  |  | -/T | 1.063(0.188,5.995) | 0.945 |  |  |
|  | g.984A>G | A/A |  | 0.869 |  |  |
|  |  | A/G | 1.144(0.434,3.019) | 0.785 |  |  |
|  |  | G/G | 1.214(0.404,3.649) | 0.729 |  |  |
|  |  | -/A | 1.962(0.570,6.755) | 0.286 |  |  |
|  |  | -/G | 1.063(0.188,5.995) | 0.945 |  |  |
|  | g.997C>T/G | C/C |  | 0.921 |  |  |
|  |  | C/G | 0.000 | 1.000 |  |  |
|  |  | G/G | 0.000 | 1.000 |  |  |
|  |  | -/C | 0.000 | 1.000 |  |  |
|  |  | -/G |  |  |  |  |
|  | g.1758G>A/T | A/G |  | 0.872 |  |  |
|  |  | G/G | 0.000 | 0.999 |  |  |
|  |  | -/G | 1.480(0.594,3.691) | 0.400 |  |  |
|  |  | -/A | 0.000 | 1.000 |  |  |
|  | g.1846G>A | A/G |  | 0.784 |  |  |
|  |  | G/G | 0.000 | 0.999 |  |  |
|  |  | -/G | 1.630(0.646,4.112) | 0.301 |  |  |
|  |  | -/A | 0.000 | 0.999 |  |  |
|  | g.2850C>T | C/C |  | 0.839 |  |  |
|  |  | C/T | 1.600(0.172,14.904) | 0.680 |  |  |
|  |  | T/T | 2.042(0.238,17.544) | 0.515 |  |  |
|  |  | -/C | 1.750(0.084,36.287) | 0.718 |  |  |
|  |  | -/T | 2.882(0.297,27.974) | 0.361 |  |  |
|  | g.2988G>A | A/G |  | 0.836 |  |  |
|  |  | G/G | 0.833(0.170,4.078) | 0.822 |  |  |
|  |  | -/G | 1.500(0.598,3.763) | 0.388 |  |  |
|  |  | -/A | 0.000 | 1.000 |  |  |
|  |  | A/A |  |  |  |  |
|  | g.3384A>C | A/A |  | 0.723 |  |  |
|  |  | A/C | 1.430(0.618,3.306) | 0.403 |  |  |
|  |  | C/C | 0.797(0.157,4.039) | 0.784 |  |  |
|  |  | -/A | 1.918(0.656,5.610) | 0.234 |  |  |
|  |  | -/C | 0.877(0.094,8.155) | 0.908 |  |  |
|  | g.3435C>A | C/C |  | 0.723 |  |  |
|  |  | -/C | 1.430(0.618,3.306) | 0.403 |  |  |
|  |  | C/A | 0.797(0.157,4.039) | 0.784 |  |  |
|  |  | A/A | 1.918(0.656,5.610) | 0.234 |  |  |
|  |  | -/A | 0.877(0.094,8.155) | 0.908 |  |  |
|  | g.4172C>T/G | C/C |  | 0.425 |  |  |
|  |  | -/C | 0.691(0.289,1.713) |  |  |  |
|  |  | C/T |  |  |  |  |
|  |  | C/G |  |  |  |  |
|  |  | -/T |  |  |  |  |
|  | g.4180G>C | C/C |  | 0.595 |  |  |
|  |  | C/G | 2.679(0.547,13.120) | 0.224 |  |  |
|  |  | G/G | 1.711(0.348,8.419) | 0.509 |  |  |
|  |  | -/C | 1.875(0.134,26.320) | 0.641 |  |  |
|  |  | -/G | 3.333(0.554,17.212) | 0.198 |  |  |
|  | full-gene-deletion | fullGene/fullGene | 1.448(0.584,3.590) | 0.425 |  |  |
|  |  | deletion/fullGene |  |  |  |  |
| CYP3A4 | c.1026+12G>A | A/A |  | 0.423 |  |  |
|  |  | A/G | 0.361(0.072,1.820) | 0.217 |  |  |
|  |  | G/G | 0.353(0.074,1.697) | 0.194 |  |  |
| CYP3A5 | c.-253-1G>A | A/A |  | 0.755 |  |  |
|  |  | A/G | 2.196(0.251,19.219) | 0.477 |  |  |
|  |  | G/G | 1.909(0.222,16.391) | 0.556 |  |  |
| CYP4F2 | c.1297G>A | A/A |  | 0.334 |  |  |
|  |  | A/G | 0.491(0.165,1.461) | 0.201 |  |  |
|  |  | G/G | 0.458(0.158,1.322) | 0.149 |  |  |
| DRD2 | c.-585A>G | A/A |  | 0.746 |  |  |
|  |  | A/G | 1.161(0.518,2.600) | 0.717 |  |  |
|  |  | G/G | 1.900(0.329,10.973) | 0.473 |  |  |
| EPHX1 | c.337T>C | C/C |  | 0.818 |  |  |
|  |  | C/T | 1.227(0.375,4.018) | 0.735 |  |  |
|  |  | T/T | 1.462(0.419,5.110) | 0.551 |  |  |
|  | c.416A>G | A/A |  | 0.729 |  |  |
|  |  | A/G | 1.422(0.597,3.390) | 0.427 |  |  |
|  |  | G/G | 0.000 | 1.000 |  |  |
| LDLR | c.*666T>C | C/C |  | 0.203 |  |  |
|  |  | C/T | 1.895(0.896,4.008) | 0.094 |  |  |
|  |  | T/T | 0.834(0.169.4.113) | 0.824 |  |  |
| LTA4H | c.-1400C>T | C/C |  | 0.112 |  |  |
|  |  | C/T | 1.697(0.580,4.967) | 0.334 |  |  |
|  |  | T/T | 3.094(1.007,9.509) | 0.049 |  |  |
| LTC4S | c.-444A>C | A/A |  | 0.988 |  |  |
|  |  | A/C | 1.025(0.422,2.488) | 0.957 |  |  |
|  |  | C/C | 1.195(0.120,11.927) | 0.879 |  |  |
| MT-RNR1 | m.1494C>T | C/C | / | / |  |  |
|  |  | C/T |  |  |  |  |
|  |  | T/T |  |  |  |  |
|  | m.1555A>G | A/A | / | / |  |  |
|  |  | A/G |  |  |  |  |
|  |  | G/G |  |  |  |  |
| NAT2 | c.282C>T | C/C |  | 0.996 |  |  |
|  |  | C/T | 1.033(0.469,2.276) | 0.936 |  |  |
|  |  | T/T | 1.004(0.313,3.222) | 0.994 |  |  |
|  | c.341T>C | C/C |  | 0.661 |  |  |
|  |  | C/T | 0.735(0.185,2.917) |  |  |  |
|  |  | T/T |  |  |  |  |
|  | c.481C>T | C/C |  | 0.661 |  |  |
|  |  | C/T | 1.361(0.343,5.401) |  |  |  |
|  |  | T/T |  |  |  |  |
|  | c.590G>A | A/A |  | 0.915 |  |  |
|  |  | A/G | 0.000 | 0.999 |  |  |
|  |  | G/G | 0.000 | 0.999 |  |  |
|  | c.803G>A | A/A |  | 0.429 |  |  |
|  |  | A/G | 0.607(0.176,2.092) |  |  |  |
|  |  | G/G |  |  |  |  |
|  | c.857G>A | A/A |  | 0.331 |  |  |
|  |  | A/G | 0.722(0.312,1.670) | 0.447 |  |  |
|  |  | G/G | 3.370(0.453,25.066) | 0.235 |  |  |
| NOS1AP | c.178-13122C>T | C/C |  | 0.971 |  |  |
|  |  | C/T | 1.105(0.488,2.499) | 0.811 |  |  |
|  |  | T/T | 1.077(0.398,2.915) | 0.884 |  |  |
| NUDT15 | c.52G>A | A/A |  | 0.882 |  |  |
|  |  | A/G | 1.189(0.120,11.771) |  |  |  |
|  |  | G/G |  |  |  |  |
|  | c.55_56insGAGTCG | GAGTCG/GAGTCG |  | 0.370 |  |  |
|  |  | -/GAGTCG | 0.557(0.155,2.002) |  |  |  |
|  |  | -/- |  |  |  |  |
|  | c.415C>T | C/C |  | 1.000 |  |  |
|  |  | C/T | 1.000(0.413,2.421) | 1.000 |  |  |
|  |  | T/T | 0.000 | 0.999 |  |  |
|  | c.416G>A | G/G |  | 1.000 |  |  |
|  |  | A/G | 0.000 |  |  |  |
|  |  | A/A |  |  |  |  |
| OPRM1 | c.118A>G | A/G |  | 0.491 |  |  |
|  |  | A/A | 1.098(0.515,2.340) | 0.808 |  |  |
|  |  | G/G | 2.252(0.590,8.591) | 0.235 |  |  |
| POLG | c.1399G>A | G/G | / | / |  |  |
|  |  | G/A |  |  |  |  |
|  |  | A/A |  |  |  |  |
| PPARG | c.34C>G | C/C |  | 0.728 |  |  |
|  |  | C/G | 0.825(0.279,2.437) |  |  |  |
|  |  | G/G |  |  |  |  |
| SCN1A | c.603-91G>A | A/A |  | 0.773 |  |  |
|  |  | A/G | 0.989(0.3902.509) | 0.982 |  |  |
|  |  | G/G | 0.731(0.250,2.138) | 0.567 |  |  |
| SCN2A | c.56G>A | A/A |  | 0.166 |  |  |
|  |  | A/G | 0.091(0.007,1.168) | 0.066 |  |  |
|  |  | G/G | 0.149(0.013,1.695) | 0.125 |  |  |
|  | c.971-32A>G | A/A |  | 0.715 |  |  |
|  |  | A/G | 0.823(0.288,2.349) |  |  |  |
|  |  | G/G |  |  |  |  |
| SLC22A1 | c.1222A>C/G | A/A |  | 0.751 |  |  |
|  |  | A/G | 0.696(0.162,2.987) | 0.626 |  |  |
|  |  | G/G | 0.592(0.139,2.517) | 0.477 |  |  |
| SLC22A2 | c.808T>G | T/T |  | 0.429 |  |  |
|  |  | G/T | 0.475(0.155,1.458) | 0.193 |  |  |
|  |  | G/G | 0.000 | 0.999 |  |  |
| SLC47A1 | c.922-158G>A | G/G |  | 0.083 |  |  |
|  |  | G/A | 0.785(0.302,2.041) | 0.620 |  |  |
|  |  | A/A | 2.004(0.740,5.428) | 0.171 |  |  |
| SLCO1B1 | c.521T>C | T/T |  | 0.005 | 3.265(1.181,9.028) | 0.023 |
|  |  | C/C, C/T | 3.167(1.413,7.096) |  |  |  |
| STXBP1 | c.922A>T | A/A | / | / |  |  |
|  |  | A/T |  |  |  |  |
|  |  | T/T |  |  |  |  |
| TPMT | c.719A>G/C | A/A | / | 0.881 |  |  |
|  |  | A/G | 0.577(0.067,4.942) | 0.615 |  |  |
|  |  | G/G | 0.000 | 1.000 |  |  |
| UGT1A | c.*211T>C | C/C |  | 0.208 |  |  |
|  |  | C/T | 0.425(0.165,1.096) | 0.077 |  |  |
|  |  | T/T | 0.000 | 1.000 |  |  |
|  | c.*339G>C | C/C |  | 0.208 |  |  |
|  |  | C/G | 0.425(0.165,1.096) | 0.077 |  |  |
|  |  | G/G | 0.000 | 1.000 |  |  |
| UGT1A1 | c.-53_-52TA | TA/TA |  |  |  |  |
|  | c.211G>A | A/A |  | 0.179 |  |  |
|  |  | A/G | 0.275(0.061,1.239) | 0.093 |  |  |
|  |  | G/G | 0.250(0.057,1.087) | 0.065 |  |  |
|  | c.-364C>T | C/C |  | 0.267 |  |  |
|  |  | C/T | 1.063(0.453,2.492) | 0.889 |  |  |
|  |  | T/T | 7.556(0.660,86.482) | 0.104 |  |  |
| UGT1A4 | c.142T>G/A | G/G |  | 0.937 |  |  |
|  |  | G/T | 0.000 | 0.999 |  |  |
|  |  | T/T | 0.000 | 0.999 |  |  |
| UGT2B15 | c.253T>G | G/G |  | 0.053 |  |  |
|  |  | G/T | 3.667(1.179,11.404) | 0.025 |  |  |
|  |  | T/T | 4.200(1.234,14.294) | 0.022 |  |  |
| VKORC1 | c.174-136C>T | C/C |  | 0.997 |  |  |
|  |  | C/T | 0.000 | 1.000 |  |  |
|  |  | T/T | 0.000 | 1.000 |  |  |
|  | c.-1639G>A | A/G |  | 0.997 |  |  |
|  |  | G/G | 1.043(0.358,3.038) | 0.939 |  |  |
|  |  | A/A | 0.000 | 1.000 |  |  |
| G6PD | c.95A>G | A/A |  | / |  |  |
|  | c.196T>A | T/T |  |  |  |  |
|  | c.202G>A | G/G |  |  |  |  |
|  | c.392G>T | G/G |  |  |  |  |
|  | c.487G>A | G/G |  |  |  |  |
|  | c.493A>G | A/A |  |  |  |  |
|  | c.517T>C | T/T |  |  |  |  |
|  | c.519C>T | C/C |  |  |  |  |
|  | c.563C>T | C/C |  |  |  |  |
|  | c.592C>T | C/C |  |  |  |  |
|  | c.871G>A | G/G |  |  |  |  |
|  | c.1004C>T | C/C |  |  |  |  |
|  | c.1024C>T | C/C |  |  |  |  |
|  | c.1360C>T | C/C |  |  |  |  |
|  | c.1376G>T | G/G |  |  |  |  |
|  | c.1388G>A | G/G |  |  |  |  |
| GRIK4 | c.83-10039T>C | C/C |  | 0.835 |  |  |
|  |  | C/T | 1.264(0.572,2.794) | 0.563 |  |  |
|  |  | T/T | 0.948(0.189,4.759) | 0.948 |  |  |
| HLA-A | *3101 | positive |  | 0.051 |  |  |
|  |  | negative | 2.823(0.995,8.009) |  |  |  |
| HLA-B | *1502 | positive |  | 0.432 |  |  |
|  |  | negative | 0.429(0.052,3.542) |  |  |  |
|  | *5801 | positive |  | 0.554 |  |  |
|  |  | negative | 0.626(0.133,2.956) |  |  |  |
| HTR1A | c.-1019G>C | C/C |  | 0.744 |  |  |
|  |  | C/G | 0.994(0,464,2.130) | 0.988 |  |  |
|  |  | G/G | 0.568(0.118,2.733) | 0.481 |  |  |
| IFNL4 | g.1332A>C | A/A |  | 0.470 |  |  |
|  |  | A/C | 0.000 | 0.999 |  |  |
|  |  | C/C | 0.000 | 0.999 |  |  |
|  | g.5710G>A | A/A |  | 0.420 |  |  |
|  |  | A/G | 0.249(0.032,1.971) | 0.188 |  |  |
|  |  | G/G | 0.000 | 0.999 |  |  |
| ITPA | c.94C>A/G | A/A |  | 0.998 |  |  |
|  |  | A/C | 0.000 | 0.999 |  |  |
|  |  | C/C | 0.000 | 0.999 |  |  |
|  | c.124+21A>C | A/A |  | 1.000 |  |  |
|  |  | A/C | 0.000 |  |  |  |
|  |  | C/C |  |  |  |  |

Abbreviations: AST, Aspartate Aminotransferase; ALT, Alanine Aminotransferase;

ALP, Alkaline Phosphatase; GGT, Gamma-Glutamyl Transferase; TP, g/L, Total Protein;

ALB: Albumin; GLB: Globulin; TBIL: Total Bilirubin; DBIL, Direct Bilirubin; IBIL, Indirect Bilirubin; TBA, Total Bile Acids; ChE, Cholinesterase; TC, Total Cholesterol; TG, Triglycerides; HDL, High-Density Lipoprotein; LDL, Low-Density Lipoprotein; B12, Vitamin B12;HCY, Homocysteine.
